# HIV-1-Specific IgA Monoclonal Antibodies from an HIV-1 Vaccinee Mediate Galactosylceramide Blocking and Phagocytosis

**DOI:** 10.1128/JVI.01552-17

**Published:** 2018-03-14

**Authors:** Saintedym Wills, Kwan-Ki Hwang, Pinghuang Liu, S. Moses Dennison, Matthew Zirui Tay, Xiaoying Shen, Justin Pollara, Judith T. Lucas, Robert Parks, Supachai Rerks-Ngarm, Punnee Pitisuttithum, Sorachai Nitayapan, Jaranit Kaewkungwal, Rasmi Thomas, Jerome H. Kim, Nelson L. Michael, Merlin L. Robb, Mike McRaven, David C. Montefiori, Thomas J. Hope, Hua-Xin Liao, M. Anthony Moody, Guido Ferrari, Barton F. Haynes, S. Munir Alam, Mattia Bonsignori, Georgia D. Tomaras

**Affiliations:** a Duke Human Vaccine Institute, Duke University Medical Center, Durham, North Carolina, USA; b Department of Medicine, Duke University Medical Center, Durham, North Carolina, USA; c Department of Immunology, Duke University Medical Center, Durham, North Carolina, USA; d Department of Molecular Genetics and Microbiology, Duke University Medical Center, Durham, North Carolina, USA; e Department of Surgery, Duke University Medical Center, Durham, North Carolina, USA; f Department of Pediatrics, Duke University Medical Center, Durham, North Carolina, USA; g Harbin Veterinary Research Institute, Chinese Academy of Agricultural Sciences, Harbin, Heilongjiang, China; h Department of Disease Control, Ministry of Public Health, Nonthaburi, Thailand; i Vaccine Trial Center, Faculty of Tropical Medicine, Mahidol University, Bangkok, Thailand; j Royal Thai Army, Armed Forces Research Institute of Medical Sciences, Bangkok, Thailand; k Center of Excellence for Biomedical and Public Health Informatics, Faculty of Tropical Medicine, Mahidol University, Bangkok, Thailand; l U.S. Military HIV Research Program, Walter Reed Army Institute of Research, Silver Spring, Maryland, USA; m Henry M. Jackson Foundation for the Advancement of Military Medicine, Bethesda, Maryland, USA; n Department of Cell and Molecular Biology, Feinberg School of Medicine, Northwestern University, Chicago, Illinois, USA; Emory University

**Keywords:** HIV-1, B cell, IgA, nonneutralizing antibodies, vaccine, monoclonal antibodies, vaccines

## Abstract

Vaccine-elicited humoral immune responses comprise an array of antibody forms and specificities, with only a fraction contributing to protective host immunity. Elucidation of antibody effector functions responsible for protective immunity against human immunodeficiency virus type 1 (HIV-1) acquisition is a major goal for the HIV-1 vaccine field. Immunoglobulin A (IgA) is an important part of the host defense against pathogens; however, little is known about the role of vaccine-elicited IgA and its capacity to mediate antiviral functions. To identify the antiviral functions of HIV-1-specific IgA elicited by vaccination, we cloned HIV-1 envelope-specific IgA monoclonal antibodies (MAbs) by memory B cell cultures from peripheral blood mononuclear cells from an RV144 vaccinee and produced two IgA clonal cell lines (HG129 and HG130) producing native, nonrecombinant IgA MAbs. The HG129 and HG130 MAbs mediated phagocytosis by monocytes, and HG129 blocked HIV-1 Env glycoprotein binding to galactosylceramide, an alternative HIV-1 receptor. These findings elucidate potential antiviral functions of vaccine-elicited HIV-1 envelope-specific IgA that may act to block HIV-1 acquisition at the portal of entry by preventing HIV-1 binding to galactosylceramide and mediating antibody Fc receptor-mediated virion phagocytosis. Furthermore, these findings highlight the complex and diverse interactions of vaccine-elicited IgA with pathogens that depend on IgA fine specificity and form (e.g., multimeric or monomeric) in the systemic circulation and mucosal compartments.

**IMPORTANCE** Host-pathogen interactions *in vivo* involve numerous immune mechanisms that can lead to pathogen clearance. Understanding the nature of antiviral immune mechanisms can inform the design of efficacious HIV-1 vaccine strategies. Evidence suggests that both neutralizing and nonneutralizing antibodies can mediate some protection against HIV in animal models. Although numerous studies have characterized the functional properties of HIV-1-specific IgG, more studies are needed on the functional attributes of HIV-1-specific IgA, specifically for vaccine-elicited IgA. Characterization of the functional properties of HIV-1 Env-specific IgA monoclonal antibodies from human vaccine clinical trials are critical toward understanding the capacity of the host immune response to block HIV-1 acquisition.

## INTRODUCTION

The RV144 ALVAC/AIDSVAX human immunodeficiency virus type 1 (HIV-1) vaccine trial demonstrated approximately 31% vaccine efficacy (60.5% vaccine efficacy through the first 12 months after vaccination) (1, 2). An immune-correlate analysis demonstrated that IgG antibodies recognizing the first and second variable loops (V1/V2) of the HIV-1 envelope (Env) glycoprotein correlated with decreased risk of HIV-1 infection, whereas high levels of Env-specific IgA antibodies correlated with increased risk of infection (3–7). From RV144 vaccine recipients, Bonsignori and coworkers have previously isolated antibody-dependent cellular cytotoxicity (ADCC)-mediating IgG monoclonal antibodies (MAbs) directed to conformational epitopes in the first constant (C1) region, the V1/V2 loops and the third variable (V3) loop of HIV-1 Env and demonstrated a synergistic effect of C1 and V1/V2 Env-specific MAbs (8–10). We have also demonstrated that vaccine-induced C1 Env-specific IgA competed IgG-mediated ADCC activity and that the plasma HIV-1 IgA/IgG ratio in vaccine recipients correlated with HIV-1 risk, suggesting that vaccine-induced IgA antibodies could have attenuated the protective effect of ADCC-mediating IgG responses through competition for the same Env binding sites (3). Similarly, interference of HIV-1 Env-specific IgA with IgG-mediated ADCC has also been suggested in the setting of HIV-1 infection (11).

However, several lines of evidence also support the notion of a potentially protective effect of IgA. In a nonhuman primate challenge study, human dimeric IgA1 protected from acquisition better than the paratope-matched dimeric IgA2 or IgG upon intrarectal simian immunodeficiency virus (SHIV) challenge (12). Several animal model (12, 13) and human (14, 15) studies also suggested that mucosal IgA could block transcytosis in mucosal tissues. HIV-1 Env-specific IgA responses have also been shown to mediate antiviral functions such as virion capture (12, 16), phagocytosis (17), and blocking of binding to the glycosphingolipid galacotsylceramide (Galcer), an alternative HIV-1 receptor (18). Further understanding of the potential antiviral functions of HIV-1 envelope-specific IgA, specifically for different isotypes and forms (monomeric, dimeric, and higher-molecular-weight forms), is needed. The isolation and characterization of the functional properties of HIV-1 Env-specific IgA monoclonal antibodies from human clinical trials will be critical to address these points.

Mucosal antibodies may impede viral transfer across mucosal surfaces by inducing viral aggregation, hindering viral movement in mucus, preventing transcytosis, engaging effector cells, or reducing intercellular penetration through the epithelia, thereby limiting access to susceptible mucosal CD4 T cells and dendritic cells (19, 20). IgA MAbs can engage effector cells through interaction of the antibody Fc with FcRα on the surfaces of effector cells. In fact, dimeric and monomeric IgA can mediate ADCC or phagocytosis by engaging FcRα on the surfaces of monocytes and polymorphonuclear leukocytes or neutrophils. In contrast, IgA cannot mediate ADCC by natural killer (NK) cells due to lack of FcRα expression on this cell type. Previous studies have reported on the potential for the duality of function for pathogen-specific IgA that is dependent on available Fc receptors; for example, Streptococcus mutans-specific IgA antibodies have been shown to promote phagocytic activities of activated human neutrophils, in which the FcRα level was upregulated, but in the absence of FcRα expression, these antibodies could mediate blocking of complement-dependent IgG-mediated phagocytosis by neutrophils (21). These data suggest that the antiviral functions of antibodies can depend on the types of effector cells present at the portal of entry for HIV and their respective FcR expression.

One strategy by which antibodies could hinder mucosal entry of HIV-1 is disruption of viral adhesion to epithelial cells. Antibodies against Galcer inhibit epithelial cell–HIV-1 virion binding (22–24), and we have demonstrated that antibodies isolated from RV144 vaccinees, both IgG and IgA, with specificities to the C1 conformational region of the HIV-1 gp120 glycoprotein could alter the Env conformation and disrupt Env-Galcer interactions (18).

In this study, we characterized two native HIV-1 Env-specific IgA MAbs, HG129 and HG130, obtained from peripheral blood memory B cells of an RV144 vaccine recipient and examined their ability to mediate antiviral activity *in vitro*. These IgA antibodies mediated monocyte phagocytosis and blocked HIV-1 Env glycoprotein to Galcer, which may be effective host defense mechanisms at the mucosal portals of entry.

## RESULTS

### Native HIV-1 IgA MAbs isolated from an HIV-1 vaccinee.

**FIG 1:**
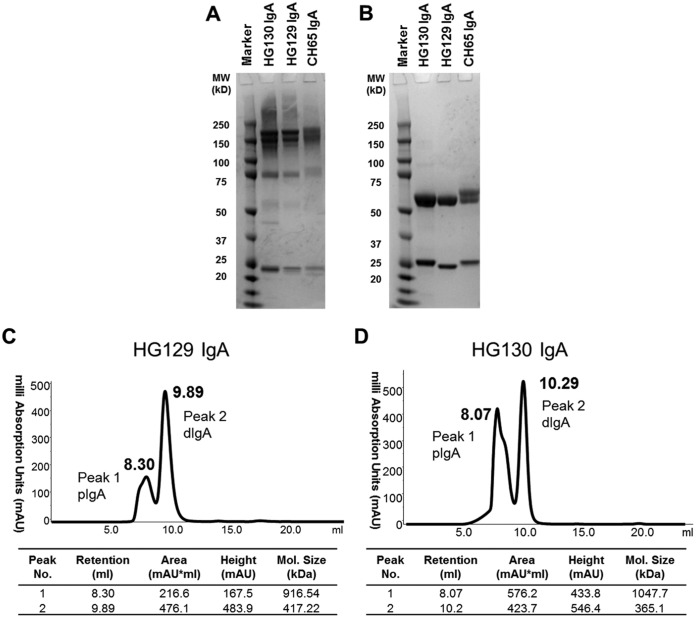
IgA monoclonal antibodies isolated from an RV144 vaccinee. (A and B) HG129 and HG130 IgA MAbs were purified with CaptureSelect IgA resin and examined by SDS-PAGE. The antibody CH65 IgA targeting influenza virus HA served as a control. Antibody forms are shown in a nonreduced gel (A) and a reduced Coomassie gel (B). (C and D) Antibodies, purified with CaptureSelect IgA resin, were analyzed by FPLC. Both dimeric (dIgA) and polymeric (pIgA) (higher forms greater than dIgA) are present in HG129 (C) and HG130 (D). The results are representative of three production batches. Molecular sizes are noted for the fractions of each antibody.

To measure vaccine-induced IgA antiviral functions, we isolated native IgA monoclonal antibodies (MAbs) from peripheral blood cultured memory B cells from an HIV-uninfected vaccinee from the RV144 clinical trial (1, 2) who had circulating IgG and IgA responses to the vaccine immunogens (i.e., 92Th023 gp120, A244 gp120, and MN gp120) (Table 1). This vaccinee did not have the HLA class II alleles, HLA-DQB1*06 and DPB1*13, reported to modulate vaccine-induced antibody responses to affect HIV-1 acquisition in RV144 (25). IgA memory B cells from peripheral blood mononuclear cells (PBMCs) were enriched by negative selection and then cultured by limiting dilution to generate monoclonal antibody cell lines. We tested the capacity of the cytokines interleukin-15 (IL-15) and B cell-activating factor (BAFF) to stimulate IgA memory B cells (26). Treatment with IL-15 and BAFF for 7 days did not impact the percentage of IgA^+^ B cells (67.1% with IL-15 and BAFF versus 68.8% without IL-15 and BAFF); however, there was a slightly increased frequency of HIV-1 Env binding IgG-negative memory B cells in the presence of cytokine stimulation (35.3% with IL-15 and BAFF versus 27.5% without IL-15 and BAFF). Supernatants from the memory B cell cultures were screened for binding to either consensus S (ConS) gp140 (group M consensus HIV-1 protein [27]) or C1 subtype AE (the binding specificity from RV144 that correlated with decreased vaccine efficacy for IgA) (3). Epstein-Barr virus (EBV)-transformed B cell lines were then derived from culture wells with the highest binding. Two IgA1 clonal cell lines, named HG129 and HG130, were selected for sequencing, analysis of variable heavy (V_H_) and light (V_L_) chains (Table 2), and characterization of antiviral functions. HG129 contained the V_H_3-30*02 gene segment paired with a V_λ_3-27*01 and had an 11-amino-acid-long heavy chain complementarity-determining region 3 (CDR H3). HG130 contained the V_H_1-8*01 paired with a V_κ_3-20*01 and had a CDR H3 of 10 amino acids (aa) in length. The V_H_ mutation frequencies of these MAbs (3.9% for HV129 and 4.8% for HG130) were low and within the expected mutation frequency for vaccine-elicited MAbs as previously reported (28). Under nonreducing conditions, sizeable portions of both the HG129 and HG130 MAbs were expressed as intact monomers (160 kDa) in the antibody preparations (29) (Fig. 1A) with a profile comparable to that of a recombinant monomeric anti-influenza virus hemagglutinin (HA) IgA control antibody (30). The higher-molecular-mass forms (i.e., >160 kDa) are multimeric forms, including dimeric IgA. Under reducing conditions, molecular mass analysis with a Coomassie gel showed heavy- and light-chain bands at approximately 55 kDa and 25 kDa, respectively (Fig. 1B). Due to the multiple forms of native IgA present, the preparations were also characterized by fast protein liquid chromatography (FPLC) with isolation of fractions for analysis in functional assays (Fig. 1C and D).

**TABLE 1:** Circulating plasma HIV-1-specific IgG and IgA binding titers^*a*^

Ig	Visit	Antibody titer (AUC)^*b*^										
		Vaccine prime gp120, 92TH023 gp120^*c*^	Vaccine boost gp120		HIV-1 gp140		CD4bs^*d*^	V3 peptides^*e*^			C1 peptide^*e*^	
			A244 gp120^*c*^	MN gp120^*c*^	1086 clade C gp140	Consensus gp140		V3 107 C	V3 B	V3 C	C1 BC	C1 AE
IgG	1	<*100*	<*100*	<*100*	<*100*	<*100*	<*100*	543	314	153.8	325	<*100*
	8	**4,168**	**4,606**	**29,300**	**30,633**	**15,769**	<*100*	440	**6,347**	**3,806**	**1,978**	**2,192**
IgA	1	<*100*	<*100*	<*100*	<*100*	<*100*	<*100*	194	<*100*	<*100*	<*100*	<*100*
	8	106	<*100*	**1,635**	**566**	185	<*100*	182	188	122	<*100*	<*100*

a Circulating plasma HIV-1-specific antibody binding titers to HIV-1 envelope proteins and peptides were measured by binding antibody multiplex assay. Antibody titers, calculated as area under the curve (AUC), are shown for HIV-specific IgG and IgA at prevaccination visit 1 and 2 weeks after the last vaccination (visit 8). Data are representative of two independent experiments.

b Visit 8 AUC values 3-fold greater than visit 1 values are in bold, and negative values of <100 are in italic.

c Env proteins contain the HIV-1 sequence without herpesvirus gD (75).

d CD4bs specificity was tested by differential binding to YU2 old core/YU2 old core D368R and RSC3/RSC3 delta 371 (76).

e Subtypes A, B, and C are noted.

**TABLE 2:** Immunogenetic properties of HG129 and HG130 monoclonal antibodies^*a*^

MAb	Isotype	V_H_	D	J_H_	HC mutation (%)	CDRH3 length (aa)	V_L_	J_L_	LC mutation (%)	CDRL3 length (aa)
HG129	IgA1	3-30*02	3-16	1	3.9	11	λ3-27*01	2	2.9	9
HG130	IgA1	1-8*01	1-1	4	4.8	10	κ3-20*01	2	6.0	8

a CDRH3, heavy-chain complementarity-determining region 3; CDRL3, light-chain complementarity-determining region 3.

### HG129 IgA MAb binding to HIV-1 envelope glycoprotein and V3 peptide.

**FIG 2:**
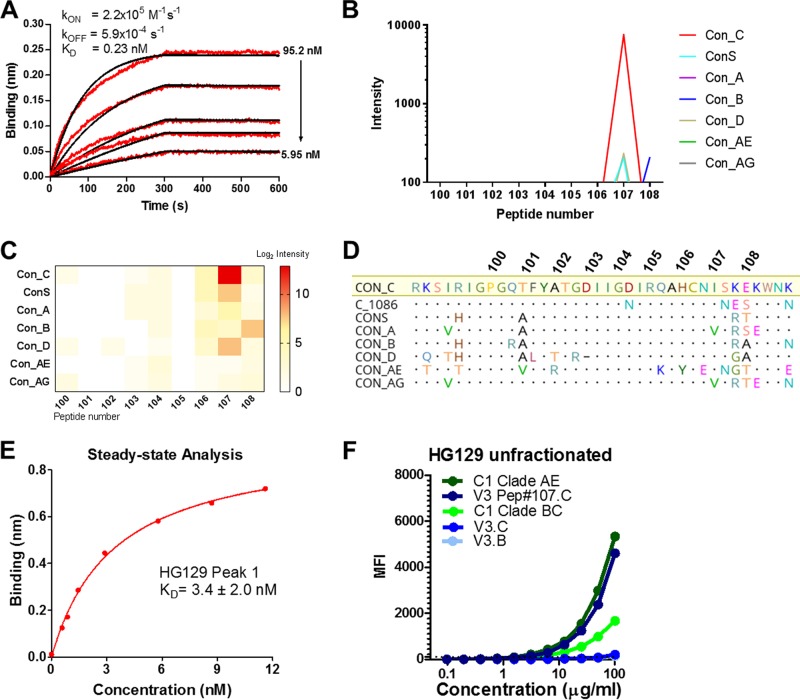
HG129 binds HIV-1 gp140 envelope glycoprotein and clade C V3 peptide with evidence of cross-reactivity. (A) Unfractionated HG129 was tested for binding to 1086.C HIV-1 gp140 envelope by biolayer interferometry (BLI). Red lines in the BLI sensogram represent specific binding time courses of 1086.C gp140 at decreasing concentrations starting at 95.2 nM for 5 dilutions (2-fold). The black lines are the best-fit curves. The mean *k*_on_, *k*_off_, and *K_d_* values obtained from the fit are shown (*n* = 3). (B and C) Binding magnitude plot (B) and heat map (C) of HG129 IgA to the clade C V3 peptide demonstrate minimal cross-clade binding (*n* = 2). (D) Sequence alignment of V3 region peptides in the array. Numbers above amino acid sequences indicate locations of center amino acids for each peptide. (E) BLI analysis of HG129 IgA binding to a clade C V3 peptide. *K_d_* values were determined by steady-state analysis using the binding response (averaged between 490 and 495 s during the association phase) of HG129 IgA peak 1 at different concentrations. (F) Binding antibody titers for unfractionated HG129 IgA were measured starting at 100 μg/ml for 12 dilutions (2-fold) for binding to HIV-1 Env peptides.

To determine the HIV-1 binding specificity for HG129, epitope mapping was performed using three independent and complementary methods: biolayer interferometry (BLI), linear peptide microarray analysis, and binding antibody multiplex assay (BAMA). In BLI analysis, the unfractionated preparation of HG129 IgA (i.e., containing a mixture of monomeric, dimeric, and polymeric antibodies) bound avidly to the HIV-1 1086 gp140 envelope glycoprotein (apparent *K_D_* [dissociation constant] = 0.23 nM) (Fig. 2A). Next, the epitope specificity and breadth of HG129 IgA were evaluated by peptide microarray analysis to determine linear binding specificities across full HIV-1 envelope sequences representing multiple subtypes (i.e., A, B, C, D, AE, and AG). Of the linear HIV specificities tested, HG129 IgA consistently bound to a subtype C V3 15-mer peptide (sequence, IRQAHCNISKEKWNK) (Fig. 2B to D) in the peptide microarray analysis and in the BLI assay with an apparent *K_D_* of 3.4 × 10^−9^ M (Fig. 2E). HG129 IgA did not bind or bound weakly to the vaccine strain antigens A244 gp120 (subtype A), 92Th023 gp120 (subtype AE), and MN gp120 (subtype B) and other HIV-1 envelope proteins (ConS gp140 [consensus group M] and 1086C envelope gp140 [subtype C]) by the fluorescent bead-based binding antibody multiplex assay. In addition to binding by peptide microarray analysis and BLI, HG129 IgA bound to a subtype C V3 peptide by the binding antibody multiplex assay (area under the curve [AUC] = 1,839) and to C1 peptide sequences (AUC = 2,098 and 558), with weaker binding to other V3 sequences (AUC = <300) (Fig. 2F). The combined data from BLI and peptide microarray analysis indicate that HG129 IgA has a strong binding avidity for the clade C V3 sequence. Notably, HG129 also bound other sequences more variably or weakly (i.e., C1 by the binding antibody multiplex assay), suggesting that HG129 also has some cross-reactive binding properties that are observed depending on the binding assay format. This suggests that conformational determinants are exposed differently among the assay formats (i.e., peptide array, protein-coupled microspheres, and in-solution binding by BLI). Another possibility is that HG129 IgA was an HIV Env cross-reactive clone existing prevaccination or otherwise not directly elicited by the vaccine. The fact that the circulating antibody responses were similar pre- and postvaccination for the V3 peptide (Table 1) provides support that MAb HG129 may be derived from a preexisting pool of B cells. However, there is also precedence for a discordance of the circulating and memory B cell responses, such that the circulating antibodies may not fully represent the memory B cell response to an antigen (31).

### HG130 IgA is a conformational gp120 antibody that interacts with the CD4 binding site.

**FIG 3:**
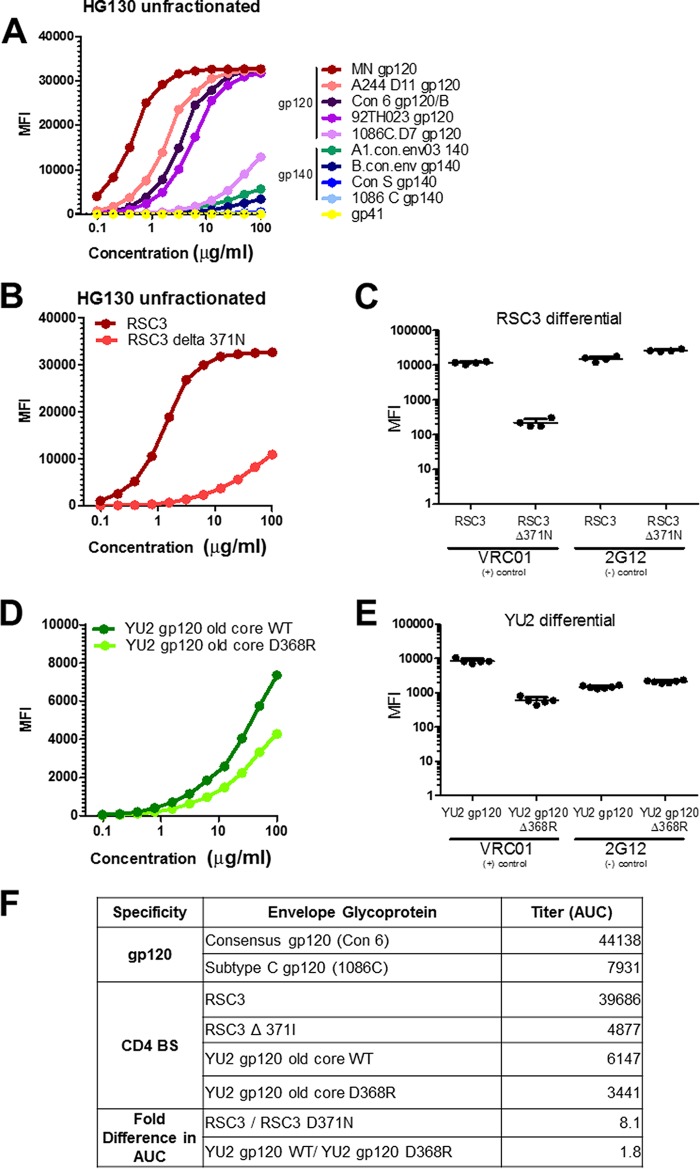
HG130 is a conformational gp120 MAb that interacts with the CD4 binding site. (A) HG130 IgA was titrated starting at 100 μg/ml for binding to a panel of cross-clade HIV-1 envelope gp120 and gp140 glycoproteins. A representative binding curve from four experiments is shown. (B and C) Differential binding of HG130 (B) and VRC01 and 2G12 (C) IgG MAbs to resurfaced stabilized core 3 (RSC3) protein and the RSC3 delta371 mutant protein that abrogates the VRC01 binding site. (D and E) Differential binding of HG130 IgA MAb (D) and VRC01 and 2G12 IgG MAbs (E) to CD4 binding site-defective protein YU2 gp120 and mutant YU2 gp120 D368R. (F) Antibody titers (*n* = 4 to 6 experiments).

HG130 bound with high titers to the vaccine strain antigens (i.e., MN gp120, A244 gp120, and 92TH023 gp120), providing evidence that HG130 was a vaccine-induced antibody (Fig. 3A). In comparison, the circulating plasma IgA response primarily targeted only one of the protein boost antigens, MN gp120 (AUC = 1,635), with no substantial reactivity to the vaccine immunogen representing the vector prime, 92TH023 gp120 (AUC = 106) and to the second protein boost antigen, A244 gp120 (AUC < 100) (Table 1). Similar to the case for HG129, these data for HG130 indicate a potential discordance in circulating versus memory B cell responses (31). To examine potential conformation epitope specificities, HG130 IgA was evaluated for reactivity with multiple subtypes of HIV-1 Env gp120 proteins (i.e., MN gp120 [subtype B], A244 gp120 [subtype AE], and 1086.C gp120 [subtype C]) (Fig. 3A). We next examined whether HG130 had properties of CD4bs antibodies. HG130 IgA bound well to the resurfaced stabilized core 3 (RSC3) protein that exposed the CD4bs epitope (AUC = 39,686), and the binding was decreased with the mutation at position 371 (RSC d371I) (AUC = 4,877) (Fig. 3B and C), indicating binding to the CD4 binding site or its vicinity. HG130 IgA also bound the YU2 gp120 wild-type protein (AUC = 6,147), and binding was reduced with the CD4 binding site-defective protein YU2 gp120 D368R (AUC = 3,441) (Fig. 3B to D). These data suggest that HG130 IgA binding involves conformational epitopes near the region on the HIV-1 Env that interacts with the host cell CD4 receptor. Since there were no substantial circulating plasma IgA CD4bs responses, HG130 IgA may be derived from a subdominant pool of antibodies targeting the CD4 binding site.

### Infected-cell recognition and virus neutralization.

**FIG 4:**
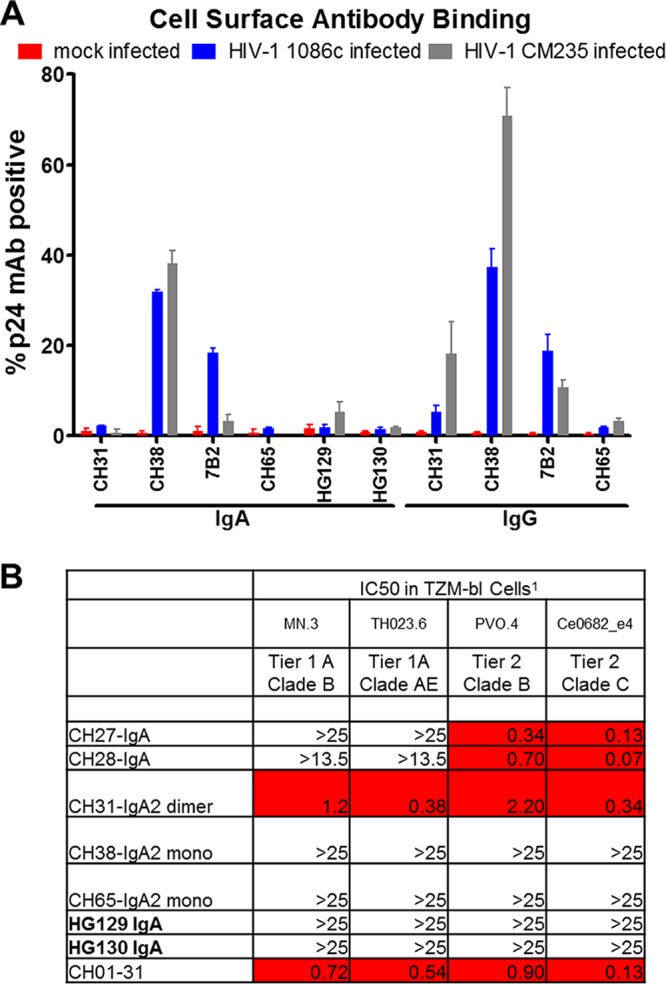
Lack of binding to HIV-1 envelope on infected cells and virus neutralization. (A) IgA (CH38 IgA and 7B2 IgA MAbs) and IgG (CH38 IgG and 7B2 IgG) positive controls bind to the surfaces of HIV-1_1086.C_-infected (blue) and HIV-1_CM235_-infected (gray) CEM NKR cells. HG129 and HG130 IgA MAbs, in addition to the negative MAb controls CH65 IgA/IgG MAbs, do not bind to the surfaces of 1086.C-infected and CM235-infected cells. The CD4bs MAb CH31 showed minimal binding as an IgG MAb and no detectable binding as an IgA MAb. Data are means and standard deviations from two independent experiments. (B) TZM-bl virus neutralization against tier 1 and tier 2 viruses are shown as antibody 50% inhibitory concentration (IC_50_) titers in μg/ml (IC_50_s of 0 to 5 are indicated in red).

Immune-correlate analysis of the RV144 vaccine trial revealed that vaccinees with plasma IgA specific to the C1 region of HIV-1 Env gp120 had a higher risk of infection (i.e., decreased vaccine efficacy) than vaccinees without C1-specific IgA antibodies (3). We determined whether the V3-specific cross-reactive HG129 IgA and the HIV-1 Env gp120-specific HG130 bound the surfaces of infected cells as a first step to evaluate the potential for blocking ADCC. We tested both HIV-1 CM235 and HIV-1 1086C to evaluate both a vaccine-related strain and an HIV-1 strain (i.e., 1086C) that HG129 bound to by BLI (Fig. 2). In contrast to the positive-control MAbs 7B2 IgA and CH38 IgA, the HG129 and HG130 IgA MAbs did not bind the surfaces of HIV-1 CM235- and HIV-1 1086C-infected cells (Fig. 4A), indicating that these antibodies do not recognize epitopes on the surfaces of infected cells and are unlikely to be capable of blocking IgG-mediated ADCC. Next, the HG129 and HG130 IgA MAbs were examined for neutralization of tier 1 and select tier 2 viruses in a TZM-bl neutralization assay (32). As a positive control, we tested the broadly neutralizing CH31 IgA MAb and two strain-specific neutralizing antibodies as IgA MAbs, CH27 and CH28 (33). There was no neutralizing activity for several HIV-1 strains tested in a TZM-bl neutralization assay (Fig. 4B).

### IgA blocking of HIV-1 1086.C gp140 Env glycoprotein binding to Galcer.

**FIG 5:**
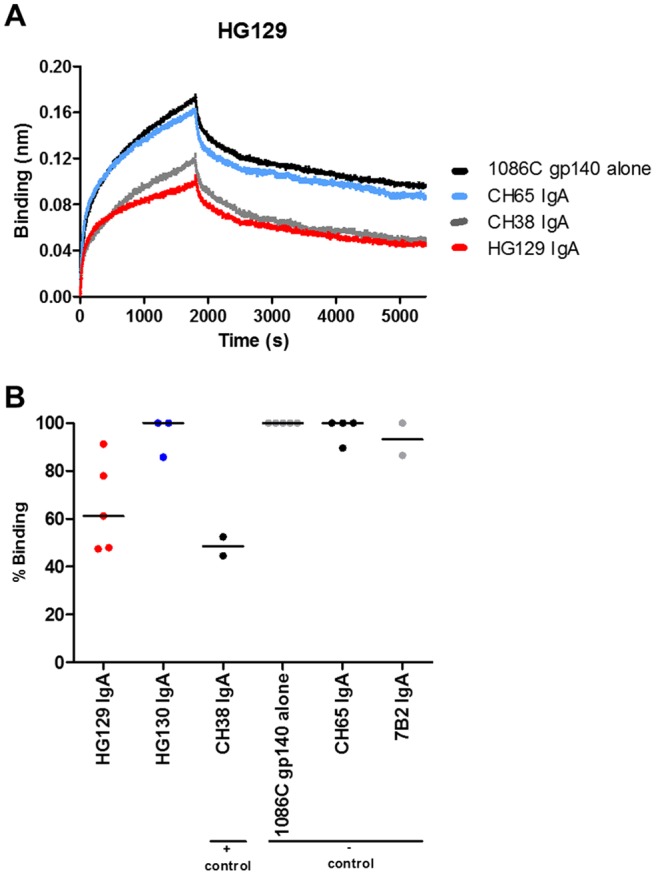
HG129 IgA blocks binding to galactosylceramide (Galcer). (A) BLI sensogram of Galcer liposome binding of Env 1086.C gp140 alone and in the presence of HG129 and positive (CH38 IgA MAb) and negative (CH65 IgA MAb) antibody controls. (B) Percent binding of Galcer to 1086.C gp140 in the presence of antibodies (*n* = 5 independent experiments). Black lines represents median values.

Since RV144 vaccinee-derived MAbs were reported to block Galcer binding of HIV-1 Env gp140 glycoproteins (18), we tested both the V3 cross-reactive HG129 IgA MAb and HIV-1 envelope gp120 HG130 MAb for their ability to block the Galcer liposome binding of HIV-1 transmitted/founder Env 1086.C gp140. Figure 5A shows the BLI sensogram of Galcer liposome binding of 1086.C gp140 alone and in the presence of CH38 IgA (positive control), CH65 IgA (negative control), and HG129 IgA antibodies. There was reduced binding of Galcer by 1086.C gp140 envelope glycoprotein in the presence of HG129 IgA compared to in the presence of negative-control IgA antibody, CH65 IgA (Fig. 5A). Figure 5B compares the percent Galcer binding of 1086.C gp140 in the presence of the IgA antibodies tested. The HG130 IgA MAb did not block Galcer binding. As noted above, Galcer binding of 1086.C gp140 was decreased by HG129 IgA (61% binding), which was almost as potent as that of the positive-control MAb CH38 IgA (48% binding) (18) (Fig. 5B).

### HG129 and HG130 IgA antibodies induce monocyte-mediated phagocytosis.

**FIG 6:**
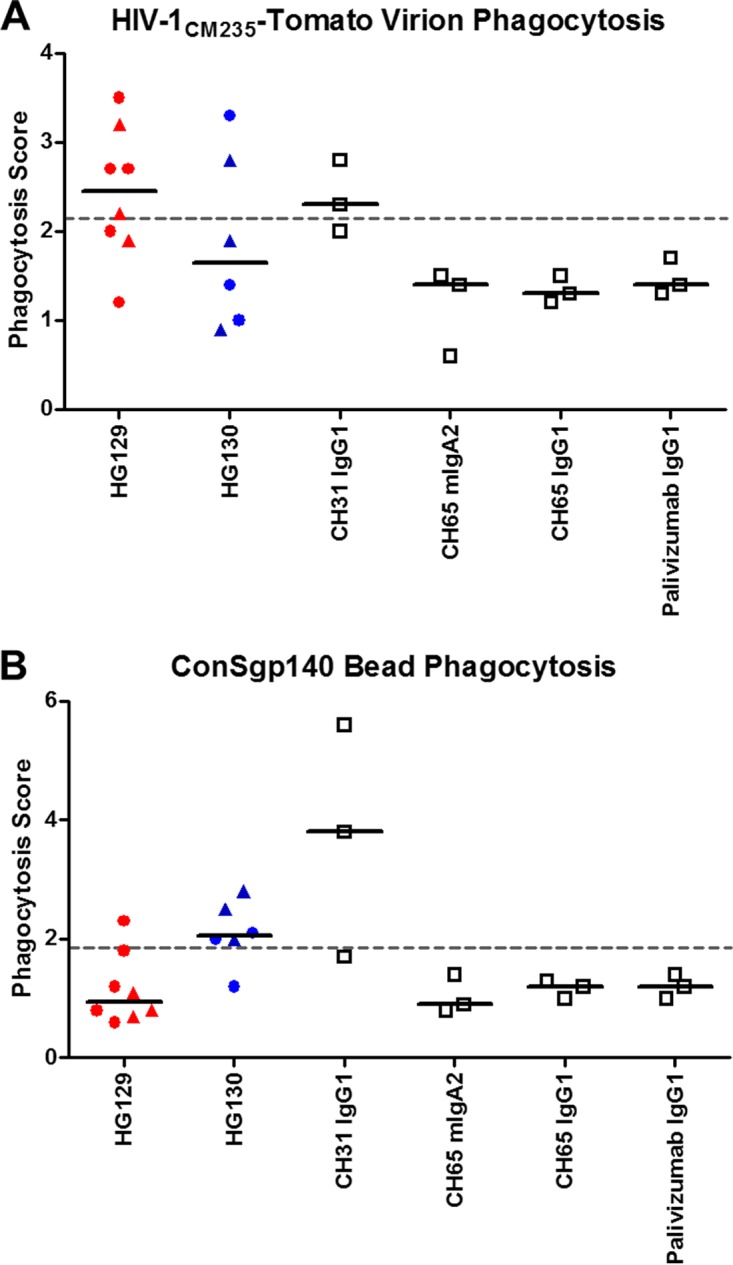
HG129 and HG130 IgA induce phagocytosis of virions or beads in primary monocytes. Phagocytosis of IgA immune-complexed infectious fluorescent HIV-1 CM235-Tomato viruses (A) and ConS gp140 Env-conjugated 1 μm fluorescent beads (B) is shown. Dimeric and polymeric fractions of HG129 and HG130 IgA antibodies were assayed in 3 independent experiments. For HG129 and HG130 IgA, each symbol corresponds to results from one antibody lot, with up to 2 replicates per antibody lot. For positive and negative controls, each symbol represents results from an independent experiment. The symbol shapes for HG129 and HG130 represent different forms of IgA (dimer, circle; polymer, triangle). Black lines indicate median results. The dashed lines indicate the average plus three standard deviations of the phagocytosis score of negative-control antibodies (CH65 mIgA2, CH65 IgG1, and palivizumab IgG1).

IgA engages in FcαRI receptor (CD89)-mediated phagocytosis of pathogens by human primary monocytes and neutrophils, especially in mucosal sites where IgA is the primary antibody isotype (34, 35). We hypothesized that HIV-1 Env-specific IgA antibodies may exert an antiviral function by engaging Fc receptor-mediated phagocytosis by monocytes. We examined antibody-mediated phagocytosis of dimeric and polymeric V3-specific cross-reactive HG129 MAb and HIV-1 envelope gp120 HG130 MAb that were purified from the unfractionated IgA by fast protein liquid chromatography. Immune complexes comprising antibodies and either fluorescent virion (HIV-1_CM235_) or bead (ConS gp140 Env-conjugated) targets were incubated with human primary monocytes to allow phagocytosis to occur. We observed that dimeric and polymeric HG129 IgA could mediate phagocytosis of HIV-1_CM235_ virions (median phagocytosis score = 2.5), while dimeric and polymeric HG130 IgA did not mediate phagocytosis of HIV-1_CM235_ virions (median phagocytosis score = 1.65) (Fig. 6A). In contrast, dimeric and polymeric HG130 IgA could mediate phagocytosis of ConS gp140 Env-coated beads (median phagocytosis score = 2.1), while dimeric and polymeric HG129 IgA did not mediate phagocytosis of ConS gp140 Env-coated beads (median phagocytosis score = 1.0) (Fig. 6B). There were no significant differences in the median phagocytosis score between the dimeric and polymeric IgAs. These results indicate that both HG129 and HG130 IgA antibodies are capable of inducing antibody-mediated phagocytosis, albeit with differences in recognition of antigen-coated beads and virus particles.

## DISCUSSION

We have identified HIV-1-specific IgA MAbs from cultured memory B cells of an RV144 vaccine recipient and demonstrated *in vitro* antiviral activity of antibodies that are of the IgA isotype and are nonneutralizing. Unlike previously identified MAbs from vaccinees, these antibodies recapitulate natural IgA forms in that they exist in polymeric, dimeric, and/or monomeric forms (reviewed in reference 36) with differences in binding avidity. Consistent with IgA forms having different avidities to antigens due to the number of antigen binding sites, we also found that dimeric IgA had greater antigen binding avidity than monomeric IgA. One IgA MAb (HG129 IgA) mediated blocking of a transmitted/founder HIV-1 Env glycoprotein binding to Galcer, an alternative HIV receptor on epithelial cells, and both IgA MAbs (HG129 and HG130 IgA) mediated phagocytosis of HIV-1 targets through engagement of human monocytes.

The immune-correlate analysis of the RV144 vaccine trial showed that certain specificities of anti-HIV-1 Env plasma (monomeric) IgA correlated with increased HIV-1 risk (i.e., decreased vaccine efficacy) by interfering with ADCC mediated by C1 region-specific IgG (3). In contrast, IgG and IgG3 responses to the V2 region of the HIV-1 Env glycoprotein correlated with decreased risk of infection (6, 7). A host genetic analysis of the RV144 case-control study revealed that the HLA class II alleles DQB1*06 and DPB1*13 modulated the impact of vaccine-induced antibody responses on HIV-1 acquisition (25). HLA class II molecules are involved in antigen presentation to CD4^+^ T cells responsible for orchestrating antipathogen immunity. The vaccinee studied here, from whom memory B cell-derived antiviral IgA antibodies were isolated, did not have the HLA-DQB1*06 allele associated with increased HIV-1 acquisition in the presence of higher levels of Env-specific IgA. Consistent with the heterogeneity observed among individuals in the specificities and magnitude of vaccine-elicited IgA and the modification of certain specificities of the HIV-1 IgA immune correlate with HIV-1 risk by certain class II alleles, it is plausible that RV144 elicited protective IgA responses in some vaccinees. We hypothesized that other vaccine-induced HIV-1 IgA antibody specificities that either were not specifically studied in the immune-correlate analysis or were present at subdominant levels may have antiviral properties. Due to limiting concentrations of circulating HIV-1 Env IgA for detailed functional studies, isolation and characterization of HIV-1 Env-specific MAbs is critical for determining potential IgA antiviral functions. HG129 and HG130 were derived from blood B cells yet mediate potentially anti-HIV responses, suggesting dual roles for Env IgA-reactive antibodies in the host response to HIV-1 that are dependent on the location of the antibody-secreting B cells.

An understudied antiviral mechanism is the role of the HIV-1 Env-Galcer interaction in establishing initial adhesion of HIV-1 virions or HIV-1-infected cells to epithelial cells (24, 37–43). Antibodies that block Galcer-Env interactions could be potentially protective at the mucosal site of HIV-1 entry. Notably, C1 conformational region-specific vaccine-induced IgG and IgA2 antibodies were shown to block the HIV-1 Env-Galcer interaction (18). We confirmed this finding and further identified and characterized HIV-1-specific IgA1 from a vaccinee that could block HIV-1 Env binding to Galcer. Therefore, the multimeric HG129 IgA could be a potential candidate for blocking the binding of virions or virus-infected cells to epithelial cells *in vivo*.

Although HG129 and HG130 were isolated from an RV144 vaccine recipient, we cannot definitively conclude that these antibodies were induced by vaccination. Circulating plasma from the RV144 recipient from whom these MAbs were derived contained V3 and C1 peptide IgG and V3 IgA binding that did not substantially differ between prevaccination and postvaccination. While HG130 bound to RV144 vaccine strain antigens and therefore was likely induced by vaccination, HG129 did not strongly bind to the vaccine strain immunogens, although it did bind to linear Env peptides and an HIV-1 envelope (not matched to the immunogen strains). These findings suggest that HG129 IgA could be derived from a preexisting pool of memory B cells that are reactive to HIV-1 antigens. Preexisting clones reactive with intestinal microbiota can cross-react with HIV Env and be expanded upon vaccination (44). We cannot exclude that some denatured or otherwise modified forms of the immunogens not represented in our *in vitro* assays engaged and expanded the HG129 clone *in vivo*. Either way, we demonstrated that HG129 IgA exerted measurable anti-HIV functions. It is possible that sequential HIV-1 immunogens could be designed to engage this low-affinity pool of B cells for greater HIV specificity and functional activity.

Stieh et al. (16) reported that Env-specific MAbs could cross-link HIV-1 virions into aggregates. The ability to form aggregated MAb-virion complexes was mediated by dimeric IgAs but not by monomeric IgA or IgG. In our study, we did not determine whether polymeric HG129 antibody could induce aggregate complexes of virions. However, our findings that polymeric IgA can block Env-Galcer interactions suggest an antiviral function of polymeric IgAs that can be harnessed by vaccination. Further studies are needed to identify if virion aggregation is a mechanism by which polymeric IgAs achieve enhanced antiviral functions.

A number of studies in humans and nonhuman primates support the potential key role for HIV-1 nonneutralizing antibodies in protection from HIV-1 acquisition (4, 6, 45–53). Although the underlying mechanisms of protection by nonneutralizing antibodies are not fully understood, antibody engagement of monocytes/macrophages, a component of the innate immune response, may play an important role in preventing HIV-1 acquisition at the mucosal site. HG129 and HG130 IgAs were both able to mediate phagocytosis of HIV-1 targets, demonstrating proof of concept that vaccine-elicited IgA is capable of engaging human immune effector cells for antiviral function. Notably, HG129 IgA was capable of mediating phagocytosis of HIV-1_CM235_ virions, indicating that its cognate epitope is present on the HIV-1 virion surface. Thus, vaccine-elicited IgA is capable of engaging virions for antiviral activity and may be relevant for vaccine-mediated protection. An HIV-1 vaccine that elicits HIV-1-specific multimeric IgA antibodies at mucosal surfaces may be able to limit viral transport across the epithelial barrier and the subsequent establishment of an infectious reservoir.

Both HG129 and HG130 IgA antibodies are nonneutralizing. Notably, although the HG130 MAb specificity includes conformational epitopes near the CD4bs, the contact sites are different from those for broadly neutralizing VRC01-like antibodies, since the D368 mutation did not fully abrogate binding and HG130 was nonneutralizing. The question remains of whether nonneutralizing IgA antibodies can protect from infection. Astronomo et al. (54) demonstrated that MAbs with known Fc effector activities but lacking potent neutralizing function were not protective when tested alone or in synergy with other antibodies. The panel of antibodies tested included these antibodies, and there was no protection against viral challenge in the *ex vivo* explant assay. Other nonneutralizing C1-C2 gp120-specific antibodies tested (CH54, CH57, CH90, and V1V2 CH58 IgG) also did not block virus infection in the *ex vivo* explant model, nor were they protective in the high-dose mucosal SHIV challenge. As the authors suggest, a limitation of the explant model is the relative frequency and distribution of monocytes, macrophages, and NK cells in vaginal tissue, which may limit timely recruitment to efficiently engage with non-broadly neutralizing antibodies to prevent initial CD4 engagement. It is possible that a low-dose intrarectal challenge could potentially tease out any *in vivo* protective activity of HG129 and HG130 or other nonneutralizing antibodies individually or in combination. Indeed, a recent study indicates that non-broadly neutralizing antibodies can be protective *in vivo* through antibody Fc interactions with FcR (55).

In summary, we have identified vaccine-elicited IgA antibodies that bind HIV-1 Env glycoproteins, block the Env gp140 glycoprotein binding to Galcer, an alternative HIV-1 receptor, and induce monocyte-mediated phagocytosis. These nonneutralizing HIV-1 IgA antibodies could be a potential candidate for blocking the binding of virus-infected cells to epithelial cells or HIV-1 transcytosis *in vivo*. Further studies, such as testing the Galcer-blocking antibody effects on inhibition of epithelial cell binding of HIV-1 virions and on cell-to-cell transmission of HIV-1 would help determine whether vaccine-elicited IgA plays a critical role in prevention of HIV transmission. Additional studies are needed to probe the specific binding region of HG129 and HG130 by generating Fabs to crystallize the MAbs in complex with gp120 core or native SOSIP trimer to evaluate the structure for phagocytosis activity and blocking Galcer. Furthermore, these antibodies could be potential candidates for passive protection trials for simian-human immunodeficiency virus (SHIV) challenge in nonhuman primates to evaluate the potential protection by HIV-specific IgA. Thus, if antibodies such as HG129 and HG130 are proven to limit transmission in passive protection trials, then design of vaccine strategies to minimize plasma Env IgA and induce mucosal Env IgA responses would be desirable.

## MATERIALS AND METHODS

### IgA memory B cell cultures.

HG129 and HG130 IgA monoclonal antibodies (MAbs) were isolated from PBMCs of an RV144 vaccine recipient. IgG-negative memory B cells were isolated from frozen PBMCs by depleting CD2-, CD3-, CD14-, CD16-, CD235a-, IgD-, and IgG-positive cells by magnetically activated cell sorting (Miltenyi Biotec, Auburn, CA). The fraction of IgG-negative memory B cells containing IgM and IgA memory B cells was resuspended in complete medium containing 2.5 μg/ml CpG ODN2006 (tlrl-2006; InvivoGen, San Diego, CA), 5 μM Chk2 kinase inhibitor (Calbiochem/EMD Chemicals, Gibbstown, NJ), and Epstein-Barr virus (EBV) (200 μl supernatant of B95-8 cells/10^4^ memory B cells) in the presence or absence of 40 ng/ml BAFF and 10 ng/ml IL-15 (PeproTech). After overnight incubation in bulk, B cells were distributed into 96-well round-bottom tissue culture plates at a density of 1,000 cells/well in the presence of irradiated (7,500 cGy) CD40 ligand-expressing L cells (5,000 cells/well). One week later, cells were screened for antibody binding to HIV-1 consensus S (ConS) gp140 Env and constant region (C1) peptide (IRQAHCNISKEKWNK), and the positive cells were set up for limiting dilution to generate monoclonal cell lines. To ensure the monoclonality of these cell lines, their variable heavy (V_H_) and light (V_L_) chains were amplified from the monoclonal cell line and analyzed for their usage of gene fragments and mutation as described below. Each cell line expressed only one V_H_ gene fragment and one V_L_ gene fragment (Table 2).

### Variable V_H_ and V_L_ chain analysis.

RNA was isolated from the human memory B cells expressing either HG129 IgA or HG130 IgA with the RNeasy minikit (Qiagen, Valencia, CA, USA) and converted to cDNA using a high-capacity cDNA reverse transcription kit (Life Technologies, Grand Island, NY, USA). Productively rearranged Ig heavy- and light-chain variable-region genes were amplified by PCR with published panels of 5′ primers for human VH (VH1-Ext–VH6-Ext), Vlambda (Vlambda1-Ext–Vlambda10-Ext), and Vkappa subgroups (56) and a 3′ primer for the VH IgA, lambda constant, and kappa constant regions. PCR products were purified using the QIAquick PCR purification kit (Qiagen) and sequences determined by the Duke University DNA Analysis facility. Sequences were analyzed using the Somatic Diversification Analysis (SoDA) software (57).

### FPLC analysis.

HG129 and HG130 IgA purified with the CaptureSelect IgA affinity matrix were analyzed and fractionated by size exclusion chromatography (SEC) on a Superdex 200 column (GE Healthcare).

### SDS-PAGE and Western blotting.

The IgA antibodies were fractionated on 4 to 12% Bis-Tris SDS-polyacrylamide gels (Invitrogen, Carlsbad, CA) under reducing (4% β-mercaptoethanol; Fisher Scientific, Fair Lawn, NJ) or nonreducing conditions, stained with Coomassie blue dye, or transferred onto nitrocellulose filters and probed with anti-J-chain MAb Jhis6 at 2 μg/ml and goat anti-human IgA conjugated with alkaline phosphatase (1:3,000 dilution; Sigma-Aldrich, Inc., St. Louis, MO) as the secondary antibody for detection.

### BLI Assay.

Biolayer interferometry (BLI) measurements were performed with a ForteBio OctetRed 384 instrument and streptavidin sensors at 25°C, and data analyses were performed with ForteBio Data Analysis 9 software. Some experiments were performed with ForteBio OctetRed 96 instrument, and the corresponding data were analyzed using ForteBio Data Analysis 7. The HIV-1 Env 1086.C gp140 binding to HG129 was carried out by immobilizing HG129 IgA antibody onto biotin-peptide M-coated streptavidin sensors and dipping into wells containing 1086.C gp140 at different concentrations (95.2, 47.6, 23.8, 11.9, and 5.95 nM). CH65 IgA immobilized sensors were used in parallel to subtract nonspecific interactions of the Env with the sensors. The specific binding curves were globally fitted to 1:1 Langmuir model to determine the on and off rates (*k*_on_ and *k*_off_, respectively) and the *K_D_* value. For HG129 IgA-V3 peptide interaction, the V3 peptide 107 (subtype C; IRQAHCNISKEKWNK)-coated sensors were prepared by dipping streptavidin sensors into wells containing V3 peptide sequences with a biotin tag (at 5 μg/ml) for 500 s. The peptide-loaded sensors were washed in phosphate-buffered saline (PBS) (pH 7.4) for 60 s before obtaining a baseline value. The binding association of HG129 was obtained by then dipping the sensor into wells with MAb concentrations ranging from 0.5 μg/ml to 10 μg/ml. The negative-control gp41 peptide LICT (biotin-GGGWGCSGKLICTT) sensors were used in parallel to subtract binding resulting from nonspecific interactions with the sensors. The apparent *K_D_* was determined using data at the end of the association phase from each available analyte concentration by steady-state analysis.

### Peptide microarray.

HIV-1 envelope epitope mapping was evaluated by peptide microarray analysis as previously described (58, 59), with modifications. Briefly, the peptide arrays contain libraries of 15-mer peptides, overlapping by 12 amino acids, spanning the whole HIV-1 gp120 Env of subtype A, B, C, and D and circulating recombinant form (CRF) AE and AG consensus sequences, as well as the group M consensus sequence, captured on customized microarrays. Three identical subarrays, each containing the full peptide library, were printed on each slide. Array slides were blocked for 1 h to reduce nonspecific binding, followed by a 2-hour incubation with 20 μg/ml IgA MAb for testing or 10 μg/ml of 7B2_IgA2 Dim (Catalent; included as positive control). The slides were subsequently incubated for 45 min with goat anti-human serum IgA (α-chain specific; Jackson ImmunoResearch) that was conjugated with DyLight 649 using a microscale antibody labeling kit (Thermo Scientific) per the manufacturer's instructions. Array slides were scanned at a wavelength of 635 nm with a GenePix 4300 scanner. Images were analyzed using GenePix software to obtain binding intensity values (median intensity of triplicates spots for each peptide).

### HIV-1 binding antibody multiplex assay.

MAb specificity for HIV-1 proteins was evaluated on a panel of HIV-1 gp120/gp140 Env glycoproteins, the linear C1 region, and the third variable-loop (V3) peptide epitopes, and V1-V2 scaffolds were measured by a custom binding antibody multiplex assay, as previously described (6, 60, 61). Positive controls included HIVIG (provided by the NIH AIDS Reagent Program) and 7B2 IgA MAb titrations and single-point HIV-1 IgG MAb controls CH58_IgG1 (9), VRC01 IgG (62), CH106 IgG (63), 2G12 IgG (64, 65), 17b IgG (66), and F105 IgG (67). Blank beads and HIV-1-negative sera were used as negative controls. HIV-1-specific antibody isotypes were detected with mouse anti-human IgA (BD Pharmingen) at 4 μg/ml, followed by washing and incubation with streptavidin-phycoerythrin (PE) (BD Pharmingen). Antibody measurements were acquired on a Bio-Plex instrument (Bio-Rad) with 21CFR Part 11 compliant software, and the readout was expressed as mean fluorescence intensity (MFI). Each test MAb was assayed at a starting concentration of 100 μg/ml and titrated by 2-fold dilutions. Results were reported as area under the curve (AUC) binding titers.

### Binding of MAbs to the surfaces of HIV-1-infected cells.

Indirect immunofluorescence staining with fluorescein isothiocyanate (FITC)-conjugated subclass-specific secondary antibodies (Sera Care/KPL, Milford, MA) was used to measure the ability of MAbs (10 μg/ml) to bind the surfaces of CEM.NKR_CCR5_ cells (NIH AIDS Reagent Program, Division of AIDS, NIAID, NIH; from Alexandra Trkola) (68), which were mock infected or infected with an infectious molecular clone virus (69) representing the 1086.C HIV-1 isolate and CM235 as previously described (8–10). Final data are reported as the mean and standard deviation of the percentage of cells positive for MAb binding within the live lymphocyte gate for mock-infected cells and the live, p24^+^ lymphocyte gate for HIV-infected cells as determined using FlowJo version 9.9.4 software (FlowJo LLC, Ashland, OR). Cells stained with FITC-conjugated secondary antibodies and no primary antibodies were used to establish the positive gates for MAb binding.

### Neutralization assay.

Neutralizing activity was measured in a TZM-bl pseudotyped virus assay as previously described (32, 33). TZM-bl cells were obtained from the NIH AIDS Research and Reference Reagent Program, as contributed by John Kappes and Xiaoyun Wu.

### Galcer blocking assay.

Galcer-liposome binding of HIV-1 gp140 glycoproteins was assayed by using the biolayer interferometry technique and a ForteBio OctetRed 96 instrument with aminopropylsilane biosensors as described previously (18). For the Galcer blocking assay, Galcer-liposome binding of HIV-1 Env 1086.C gp140 at 50 μg/ml was monitored for 30 min, and the dissociation phase was monitored for 1 h. In parallel, Galcer-liposome binding of 1086.C gp140 (50 μg/ml) incubated with a 3 molar excess of HG129 or HG130 IgA was monitored. The Galcer-liposome binding responses (after subtracting the signal found with blank sensors) at the end of a 1-h dissociation phase were averaged (over a 20-s window). The percent Galcer binding was calculated as (*B*/*A*) × 100%, where *A* is the Galcer binding response of 1086.C gp140 and *B* is the Galcer binding response of 1086.C gp140 in the presence of a 3 M excess of the antibody of interest.

### Phagocytosis assay.

Antibody-mediated phagocytosis was assayed as previously described (17). Phagocytosis potency was tested against infectious fluorescent HIV-1_CM235_ virions (70, 71) and against ConSgp140 Env-conjugated NeutrAvidin fluorescent 1-μm beads (Thermo Fisher). Blood-derived monocytes were used as phagocytes. The monocytes were isolated from peripheral blood mononuclear cells (PBMCs) of HIV-1-negative healthy donors via elutriation. PBMCs from HIV-1-negative individuals were collected with IRB approval by the Duke Medicine Institutional Review Board for Clinical Investigations. All subjects were asked for consent following 45 CFR 46, and written informed consent was obtained from all participants. Positive controls included the CD4 binding site broadly neutralizing antibody CH31 (72) in monomeric IgA2 (mIgA2) (73), IgG1, and IgG3 backbones, while negative controls included the anti-influenza virus HA antibody CH65 (30) in mIgA2 and IgG1 backbones as well as the anti-respiratory syncytial virus antibody Palivizumab IgG1.

### HLA typing.

Genotyping of HLA was performed by next-generation sequencing of full-length genes on the MiSeq Illumina platform as described previously (74).

### Accession number(s).

Nucleotide sequences for the HG129 and HG130 MAbs are deposited in GenBank with accession numbers MF939400 (V_H_) and MF939401 (V_L_) for MAb HG129 and MF939402 (V_H_) and MF939403 (V_L_) for MAb HG130.
